# Expression of RET finger protein predicts chemoresistance in epithelial ovarian cancer

**DOI:** 10.1002/cam4.32

**Published:** 2012-09-13

**Authors:** Maiko Horio, Takuya Kato, Shinji Mii, Atsushi Enomoto, Masato Asai, Naoya Asai, Yoshiki Murakumo, Kiyosumi Shibata, Fumitaka Kikkawa, Masahide Takahashi

**Affiliations:** 1 Department of Pathology, Nagoya University Graduate School of MedicineNagoya, Japan; 2 Department of Obstetrics and Gynecology, Nagoya University Graduate School of MedicineNagoya, Japan

**Keywords:** Carboplatin, chemoresistance, epithelial ovarian cancer, paclitaxel, RET finger protein

## Abstract

Resistance to platinum- and taxane-based chemotherapy is a major cause of treatment failure in ovarian cancer. Thus, it is necessary to develop a predictive marker and molecular target for overcoming drug resistance in ovarian cancer treatment. In a previous report, using an in vitro model, we found that the RET finger protein (RFP) (also known as tripartite motif-containing protein 27, TRIM27) confers cancer cell resistance to anticancer drugs. However, the significance of RFP expression in cancer patients remains elusive. In this study, we showed that RFP was expressed in 62% of ovarian cancer patients and its positivity significantly correlated with drug resistance. Consistent with clinical data, depletion of RFP by RNA interference (RNAi) in ovarian cancer cell lines, SKOV3 and HEY, significantly increased carboplatin- or paclitaxel-induced apoptosis and resulted in reduced anticancer drug resistance. In a nude mouse tumor xenograft model, inoculated RFP-knockdown ovarian cancer cells exhibited lower carboplatin resistance than control cells. These findings suggest that RFP could be a predictive marker for chemoresistance in ovarian cancer patients and also a candidate for a molecular-targeted agent.

## Introduction

Ovarian cancer is the fifth most common malignancy among women worldwide and the leading cause of gynecologic cancer-related deaths [1]. Over the past 20 years, the mortality rate of ovarian cancer has remarkably increased in Japan [2]. Approximately 225,000 new ovarian cancer cases were diagnosed worldwide, and there were approximately 140,000 deaths because of ovarian cancer in 2008 [3]. Approximately 70% of ovarian cancer patients are diagnosed at advanced stages of the disease, which is one of the causes of poor prognosis [4, 5].

Advanced-stage epithelial ovarian cancers are managed with cytoreductive surgery and chemotherapy consisting of carboplatin and paclitaxel, achieving complete clinical remission in most patients [6]. However, although the initial response rate to chemotherapy is high, most advanced-stage ovarian cancer patients relapse. In recurrent ovarian cancer, intrinsic and acquired chemoresistance is a major cause of treatment failure [7].

During primary therapy, 70% of ovarian cancer patients respond to platinum compounds; however, only 42% respond to taxanes according to the results of the Gynecologic Oncology Group (GOG) 132 study [8]. Although some characteristics such as the status of p53 mutation or upregulation of class III tubulin, annexin A3, or bcl-2 have been reported to be potential indicators of chemoresistance [9–12], there are no promising biomarkers that predict chemoresistance in ovarian cancer patients. Thus, it is important to identify new diagnostic and prognostic indicators and molecular pathways involved in drug resistance of ovarian cancer cells for developing novel therapeutic strategies.

*RFP* was first identified as a gene involved in the generation of the *RET*-transforming gene activated by DNA rearrangement [13–15]. RFP contains a tripartite motif consisting of a RING finger, B-box zinc finger, and coiled-coil domain and exhibits transcriptional repressive activity through the association with several transcriptional regulators such as enhancer of polycomb 1, Mi-2β, and retinoblastoma protein (RB1) in the nucleus [16–18]. We showed that the protein complex consisting of RFP, HDAC1, and NF-Y confers anticancer drug resistance by decreasing thioredoxin-binding protein-2 (TBP-2) expression, which inhibits thioredoxin function [19]. Clinicopathological studies revealed that positive RFP expression is a predictive marker for an unfavorable clinical outcome of endometrial cancer and colon cancer patients [19, 20]. These findings suggest that RFP is involved in tumor progression, including the acquisition of chemoresistance.

In this study, we evaluated the significance of RFP expression in epithelial ovarian cancer and showed for the first time that positive RFP expression increased in recurrent ovarian cancer, correlating with a poor outcome of the patients. The positive rate of RFP expression in platinum-resistant patients was significantly higher than that in platinum-sensitive ones. We provide evidence that RFP enhances chemoresistance of ovarian cancer cells to carboplatin and paclitaxel in culture cells and xenografts. These findings imply that RFP could be a good molecular target for ovarian cancer.

## Materials and Methods

### Patients and tissue samples

Tissue samples were obtained from patients who underwent surgical treatment at Nagoya University Hospital between 1998 and 2008 after obtaining informed consent. The patient age ranged from 26 to 79 years, with a median age of 53 years. The present series consisted of 92 ovarian carcinomas (45 serous adenocarcinomas, 10 mucinous adenocarcinomas, 13 endometrioid adenocarcinomas, and 24 clear cell carcinomas). The histological cell types were assigned according to the World Health Organization classification criteria (serous, endometrioid, and mucinous types). Clinical stage was assigned on the basis of the International Federation of Gynecology and Obstetrics (FIGO) staging system. All tissue samples were fixed in 10% formalin, embedded in paraffin, and stained with hematoxylin and eosin for histological examination. All patients were treated with platinum-based chemotherapy. They were administered paclitaxel/carboplatin (66 cases), cisplatin/vinblastine/bleomycin (seven cases), docetaxel/carboplatin (four cases), CPT-11/cisplatin (three cases), docetaxel/cisplatin (two cases), cyclophosphamide/doxorubicin/cisplatin (one case), or cisplatin/carboplatin (nine cases). Clinically, patients who respond to first-time platinum-based therapy and have a recurrence-free interval of 6 months or more are considered platinum sensitive, and those who progress during the initial platinum-based therapy or who recur within 6 months after completion of platinum-based therapy are considered platinum resistant (primary chemoresistant) [10, 21].

### Cell culture

Epithelial ovarian cancer cell lines, SKOV3 and HEY, were obtained from Memorial Sloan-Kettering Cancer Center (New York, NY), and University of Texas, MD Anderson Cancer Center (Houston, TX), respectively. Both cell lines were maintained in RPMI 1640 medium (Sigma, St. Louis, MO) supplemented with 10% fetal bovine serum (FBS), penicillin (100 U/mL), and streptomycin (100 g/mL) at 37°C in a humidified 5% CO_2_ atmosphere.

### Antibodies

Rabbit polyclonal RFP antibody has been described previously [16]. Anti-β-actin monoclonal antibody was purchased from Sigma. Alexa-conjugated secondary antibody was purchased from Molecular Probes (Carlsbad, CA). Anti-cleaved caspase 3 monoclonal antibody was purchased from Cell Signaling Technology (Danvers, MA).

### Immunohistochemical staining

Formalin-fixed, paraffin-embedded tissue sections from the ovarian cancer patients were deparaffinized and rehydrated. Slides were thoroughly rinsed with Protein Blocking Agent (UltraTech HRP Streptavidin–Biotin Detection System; Beckman Coulter, Fullerton, CA), and endogenous peroxidase was blocked with 3% hydrogen peroxide (H_2_O_2_). Antigen retrieval was performed by autoclaving at 121°C for 10 min in 0.1 mol/L citrate buffer (pH 7.0) with 0.5% NP-40 (Sigma). The sections were incubated with rabbit polyclonal RFP antibody (1 μg/mL) for 60 min followed by incubation with secondary biotinylated goat polyvalent antibody (Beckman Coulter) for 10 min. The samples were incubated with peroxidase-conjugated streptavidin for 10 min, and the reaction products were visualized using 3,3′-diaminobenzidine tetrahydrochloride and H_2_O_2_. Counterstaining was performed using hematoxylin. To evaluate RFP expression levels, the staining intensity was scored as 0 (negative), 1 (weak), 2 (medium), or 3 (strong). The extent of the area stained in the cancer tissues was scored as 0 (<10%), 1 (10–30%), 2 (30–50%), or 3 (>50%). The sum of scores for the staining intensity and staining extent was used as the staining score (0–6) for RFP.

### RNA interference

RFP siRNA (siRFP) and control siRNA (siCont) were purchased from Qiagen (Valencia, CA) and transfected into cell lines using Lipofectamine 2000 (Invitrogen, Carlsbad, CA) according to the manufacturer's instructions. The target sequences were as follows: RFP sense, 5′-TGCTCGACTGCGGCCATAAC-3′ and RFP antisense, 5′-TCGGTGCGCAGCTGCTTTAC-3′.

### Generation of cell lines stably expressing RFP short-hairpin RNA

Target sequences for short-hairpin RNA (shRNA)-mediated RFP knockdown were described previously [19]. The oligonucleotide pair was annealed and inserted into the pSIREN-RetroQ retroviral shRNA expression vector (Clontech, Palo Alto, CA). To produce retroviral supernatants, GP2-293 packaging cells were seeded in collagen type 1-coated 100-mm cell culture dishes and transfected with the pVSV-G vector and either control or RFP shRNA-containing pSIREN-RetroQ vector using Lipofectamine 2000 reagent (Invitrogen). The medium was replaced after 24 h, and virus-containing supernatants were harvested 48 h posttransfection and used for infecting SKOV3 or HEY cells. The infected cells were then selected in puromycin-containing medium for 2 days.

### Western blot analysis

Briefly, 20 μg of total cell lysates were electrophoresed on a 10–15% SDS-polyacrylamide gel and electrophoretically transferred to Immobilon membranes (Millipore, Bedford, MA). After treatment with blocking solution (5% nonfat dry milk/0.1% Tween-20/phosphate-buffered saline [T-PBS]), the membranes were incubated overnight with a recommended dilution of the following primary antibodies: rabbit polyclonal RFP antibody and anti-β-actin antibody (Sigma). The membranes were washed three times for 15 min each with 0.1% T-PBS and then incubated with the appropriate secondary antibody for 1 h. After washing with T-PBS, proteins were visualized using enhanced chemiluminescence reagent (Amersham Pharmacia Biotech, Uppsala, Sweden) followed by X-ray film exposure.

### Cell proliferation assay

The cells were seeded in triplicate in 96-well plates at a density of 2000 cells in 100 μL of RPMI-1640 containing 10% FBS at concentrations of 40 μg/mL carboplatin, 10 ng/mL paclitaxel, or anticancer drug free, and cultured for 1–3 days. Cell proliferation was evaluated by the 3-(4,5-dimethylthiazol-2-yl)-5-(3-carboxymethoxyphenyl)-2-(4-sulfophenyl)-2H-tetrazolium (MTS) assay using the Cell Titer 96 Aqueous One Solution Cell Proliferation Assay kit (Promega, Madison, WI) according to the manufacturer's instructions. Absorbance was measured at 492 nm using a microplate reader (Biotek, Winooski, VT).

### Cell viability assay

Cell viability was determined by the MTS assay using the Cell Titer 96 Aqueous One Solution Cell Proliferation Assay kit (Promega). Briefly, 5 × 10^3^ cells per well were seeded in 96-well plates and treated for either 48 h with medium containing serial concentrations of carboplatin (0–60 μg/mL) or 72 h with medium containing serial concentrations of paclitaxel (0–100 ng/mL). MTS solution (20 μL) was added to each well. The plates were incubated for an additional hour at 37°C, and the absorbance at 492 nm was recorded using a microplate reader (Biotek) to calculate the cell survival percentages.

### Fluorescence microscopy

The cells were plated on collagen type 1 (Millipore)-coated 35-mm glass base dishes (IWAKI, Tokyo, Japan) and cultured in growth media for 24 h prior to carboplatin and paclitaxel treatment. Forty-eight hours after anticancer drug treatment, the cells were fixed in 4% paraformaldehyde for 1 h at RT, washed in PBS, and blocked with 1% BSA. Cleaved caspase 3 was detected using anti-cleaved caspase 3 rabbit monoclonal antibody (1:1600). The cells were then stained with Alexa Fluor 488-conjugated anti-rabbit IgG antibody (1:1000) and 4′,6-diamidino-2-phenylindole (DAPI), and observed using a confocal microscope (Olympus, Tokyo, Japan) (DAPI, 405 nm; Alexa Fluor 488, 488 nm).

### Nude mouse tumor xenografts and carboplatin treatment

SKOV3 cells (5 × 10^6^) stably expressing shRFP or shCont in 100 μL of PBS were subcutaneously injected into the right flank of 6-week-old female nude mice (Crlj:CD1-Foxn1nu; Charles River Laboratories Japan Inc., Yokohama, Japan). When the tumor volume reached approximately 50 mm^3^, the mice inoculated with shRFP or shCont were assigned into the PBS- or carboplatin-treated group. Each group (*n* = 8–10) was administered PBS or carboplatin intraperitoneally (i.p.) four times every 2 days. The tumor volumes were measured by calipers and estimated using the following formula: volume = length × width × width × 1/2. The mice were maintained in accordance with the institutional guidelines of Nagoya University Graduate School of Medicine, and experiments were performed according to approved experimental protocols.

### Statistical analysis

Kaplan–Meier survival curves were plotted using GraphPad Prism 5.0 software (GraphPad Software, San Diego, CA) and compared using the log-rank test. The prognostic significance of positive RFP expression in relation to other clinicopathological variables was assessed using multivariate Cox proportional hazards analysis. SPSS software (SPSS Inc., Chicago, IL) was used for multivariate analysis. For data from in vivo and in vitro experiments, statistical comparisons between groups were performed using nonpaired Student's *t-*test. Differences between groups were considered significant at *P* < 0.05. Data are expressed as the mean ± SD.

## Results

### Immunohistochemical analysis of RFP expression in epithelial ovarian cancer

**Figure 1:**
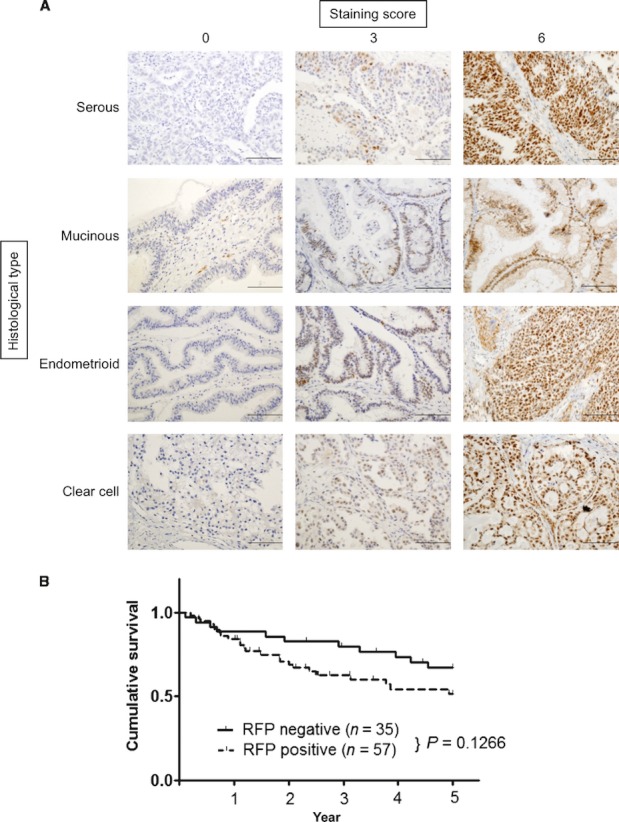
Immunohistochemical analysis of RFP in epithelial ovarian cancer. (A) RFP expression in serous, mucinous, endometrioid, and clear cell adenocarcinomas. To evaluate RFP expression, the staining intensity was scored as 0 (negative), 1 (weak), 2 (medium), or 3 (strong). The staining extent was scored as 0 (<10%), 1 (10–30%), 2 (30–50%), or 3 (>50%) in relation to the entire cancer area. The sum of scores for the staining intensity and staining extent was used as the staining score (0–6) for RFP. Staining score 0–2, negative; 3–6, positive. Representative images with staining scores 0 (left), 3 (middle), and 6 (right) are shown. Scale bars: 200 μm. (B) Kaplan–Meier survival curves of patients with epithelial ovarian cancer stratified by RFP expression. The 5-year overall survival rate: all cases (*n* = 92).

To evaluate RFP expression in ovarian cancer, formalin-fixed, paraffin-embedded tissue sections from 92 patients were immunostained with anti-RFP antibody. RFP expression was specifically detected in the nuclei of cancer cells but not in those of nonmalignant cells surrounding the cancer cells. When the staining score of RFP expression was >3 (see Materials and Methods), the specimen was classified as RFP positive (Fig. 1A). Of the 92 ovarian cancer specimens examined in this study, 57 (62.0%) were positive for RFP immunoreactivity. No significant association was found between RFP immunoreactivity and any clinicopathological parameters, including age, FIGO stage, and histological type (Table 1). Positive RFP immunoreactivity was significantly correlated with cancer recurrence after completion of platinum-based therapy (*P* = 0.0431, Table 1). The 5-year overall survival (OS) curve of the ovarian cancer patients with respect to RFP expression showed a trend toward poor prognosis for the patients with positive RFP immunoreactivity, although it was not statistically significant (*P* = 0.1266, Fig. 1B).

**Table 1:** Association between RFP expression and clinicopathologic factors in patients with ovarian cancer

Variables	Number of patients	RFP(−)	RFP(+)	*P*
Age				
<60	65	27 (41.5%)	38 (58.5%)	0.2840
≥60	27	8 (29.6%)	19 (70.4%)	
FIGO stage				
I	29	13 (44.8%)	16 (55.2%)	0.3632
II∼IV	63	22 (34.9%)	41 (65.1%)	
Histological type				
Nonclear	68	29 (42.6%)	39 (57.4%)	0.1258
Clear cell	24	6 (25.0%)	18 (75.0%)	
Recurrence				
−	42	21 (50.0%)	21 (50.0%)	0.0431
+^1^	50	14 (28.0%)	36 (72.0%)	
Time before recurrence (platinum-free interval)				
≥6 months	60	30 (50.0%)	30 (50.0%)	0.0015
<6 months^1^	32	5 (15.6%)	27 (84.4%)	

RFP, RET finger protein; FIGO, International Federation of Gynecology and Obstetrics.

1 Including PD (progressive disease).

Next, we examined whether RFP expression predicts chemoresistance in ovarian cancer. The percentage of RFP-positive cases was significantly greater in the patients with <6-month recurrence (drug resistance, 84.4%) than in those with ≥6-month recurrence (drug sensitive, 15.6%) (*P* = 0.0015, Table 1). We further analyzed whether RFP-mediated chemoresistance is related to the histological types of ovarian cancer. Significant association between RFP expression and chemoresistance was observed in serous and clear cell carcinomas (*P* = 0.0202 and 0.0285, respectively) but not in mucinous and endometrial carcinomas (Table S1).

In addition, we performed multivariate OS analysis, in relation to age, FIGO stage, histological type, and RFP immunoreactivity. Cox proportional hazards analysis revealed that the FIGO stage, but not RFP immunoreactivity, was an independent prognostic factor (Table S2).

### RFP knockdown enhances chemosensitivity in ovarian cancer cell lines

**Figure 2:**
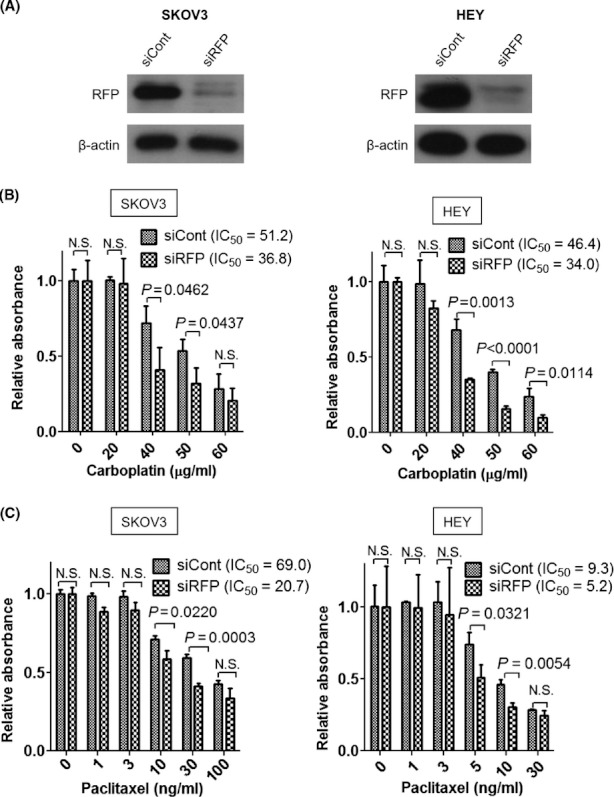
RFP knockdown enhances chemosensitivity in ovarian cancer cell lines. (A) Control siRNA (siCont) and RFP siRNA (siRFP) were transfected into SKOV3 and HEY cells 72 h before the assays. Total cell lysates from each cell line were subjected to Western blotting with anti-RFP or anti-β-actin antibody. (B) and (C) SKOV3 and HEY cells were transfected with siCont or siRFP and incubated for 48 h. The cells were treated with the indicated doses of carboplatin (B) for 48 h or paclitaxel (C) for 72 h. Cell viability was measured using the MTS assay.

To determine whether RFP expression is related to anticancer drug sensitivity, epithelial ovarian cancer cell lines, SKOV3 and HEY, were transfected with siRFP or siCont and treated with carboplatin or paclitaxel. siRFP transfection effectively reduced RFP expression compared with siCont transfection (Fig. 2A). To investigate the effects of RFP knockdown on carboplatin sensitivity, the MTS assay was performed using siRNA-transfected SKOV3 and HEY cells. As shown in Figure 2B, siRFP transfection sensitized SKOV3 and HEY cells to carboplatin, showing that the half maximal inhibitory concentration (IC_50_) for carboplatin in both the cell lines was decreased compared with that in the control (1.39-fold and 1.36-fold decrease in IC_50_, respectively). We then examined the effect of RFP knockdown on cytotoxic activity of paclitaxel, which is generally used for chemotherapy of ovarian cancer in combination with carboplatin. Figure 2C shows that RFP depletion also sensitized SKOV3 and HEY cells to paclitaxel, as observed for carboplatin (3.33-fold and 1.79-fold decrease in IC_50_, respectively).

**Figure 3:**
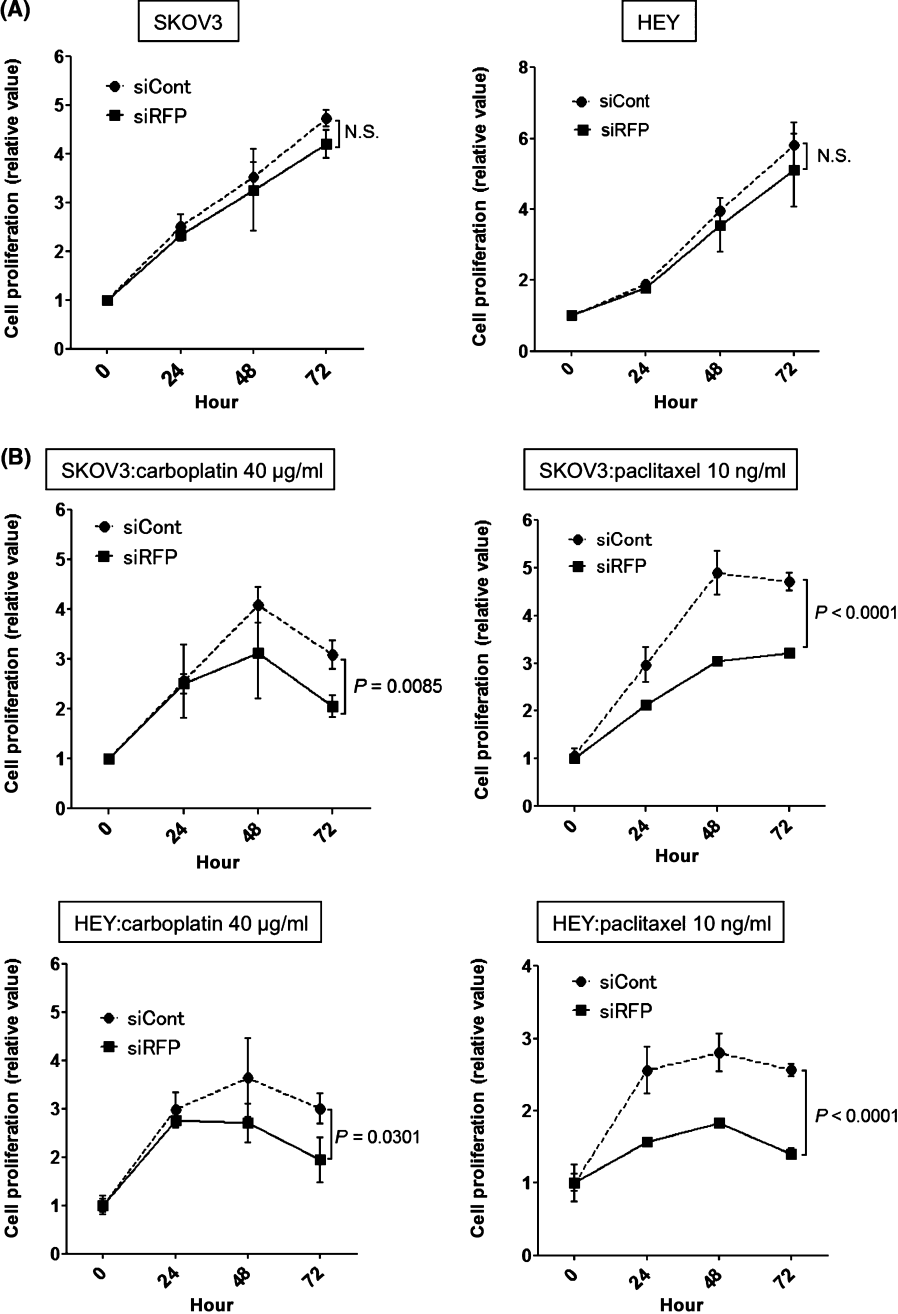
Effects of RFP knockdown on cancer cell proliferation. Time point MTS assay of SKOV3 and HEY cells transfected with each siRNA. (A) The assays were performed without anticancer drugs. (B) SKOV3 cells were cultured in RPMI containing 40 μg/mL of carboplatin (left) or 10 ng/mL of paclitaxel (right) (top panels); HEY cells were cultured in RPMI containing 40 μg/mL of carboplatin (left) or 10 ng/mL of paclitaxel (right) (bottom panels).

In the cell proliferation assay, siRFP transfection did not significantly affect SKOV3 and HEY cell proliferation (Fig. 3A). However, when SKOV3 and HEY cells were treated with carboplatin (40 μg/mL) or paclitaxel (10 ng/mL), RFP knockdown significantly inhibited cancer cell proliferation (Fig. 3B). These results showed that reduction of RFP expression enhances the therapeutic effect of anticancer drugs in vitro.

### RFP knockdown increases carboplatin- and paclitaxel-induced apoptosis of ovarian cancer cell lines

**Figure 4:**
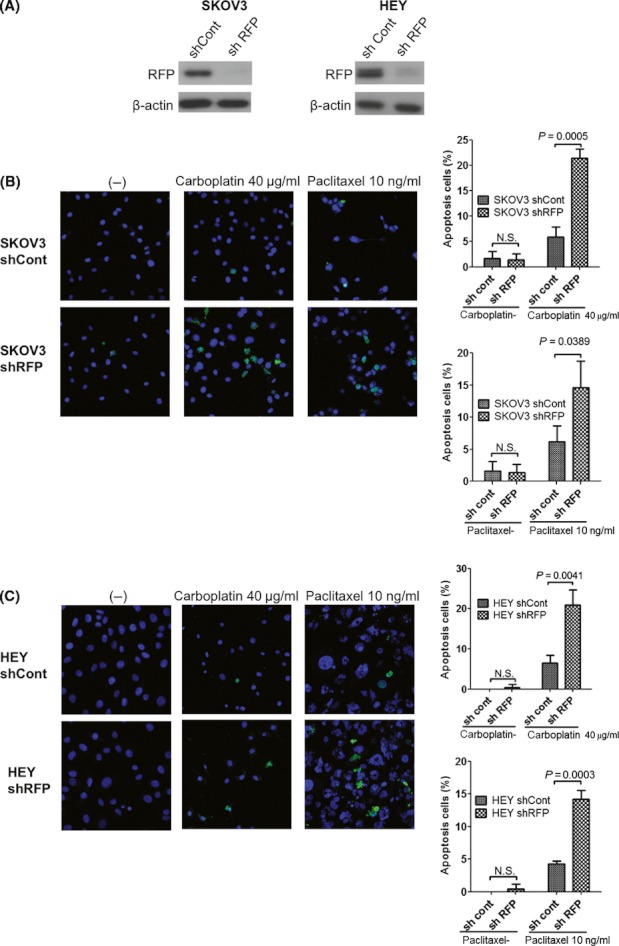
Effect of RFP knockdown on apoptosis of carboplatin- and paclitaxel-treated ovarian cancer cells. (A) SKOV3 or HEY cells were infected with retroviruses carrying control or RFP-targeting shRNA. Total cell lysates from each cell line were subjected to Western blotting with anti-RFP or anti-β-actin antibody. Representative images of SKOV3 (B) and HEY (C) cells infected with retroviruses carrying shCont or shRFP. These cells were treated with carboplatin or paclitaxel at the indicated concentration for 48 h. The cells were stained by anti-cleaved caspase 3, and positive cells were counted under ×200 magnification. Percentages of cleaved caspase 3-positive cells are shown in right panels. Total 200 cells were counted. Bars, SD. *P* < 0.05 was considered statistically significant. N.S., not significant.

To better understand the mechanisms of RFP depletion-mediated enhancement of anticancer drug sensitivity, we analyzed the effect of RFP knockdown on anticancer drug-induced apoptosis of ovarian cancer cells. Infection by shRFP-expressing retrovirus effectively reduced RFP expression compared with that by control virus (Fig. 4A). To count apoptotic cells after carboplatin or paclitaxel treatment, the cells were stained with anti-cleaved caspase 3 antibody. RFP knockdown significantly increased the number of apoptotic cells after carboplatin or paclitaxel treatment in both SKOV3 (Fig. 4B) and HEY (Fig. 4C) cells, indicating that an increase in induced apoptosis by carboplatin or paclitaxel treatment could be the major cause of enhanced chemotherapeutic effects by RFP knockdown.

### RFP depletion confers carboplatin resistance in vivo

**Figure 5:**
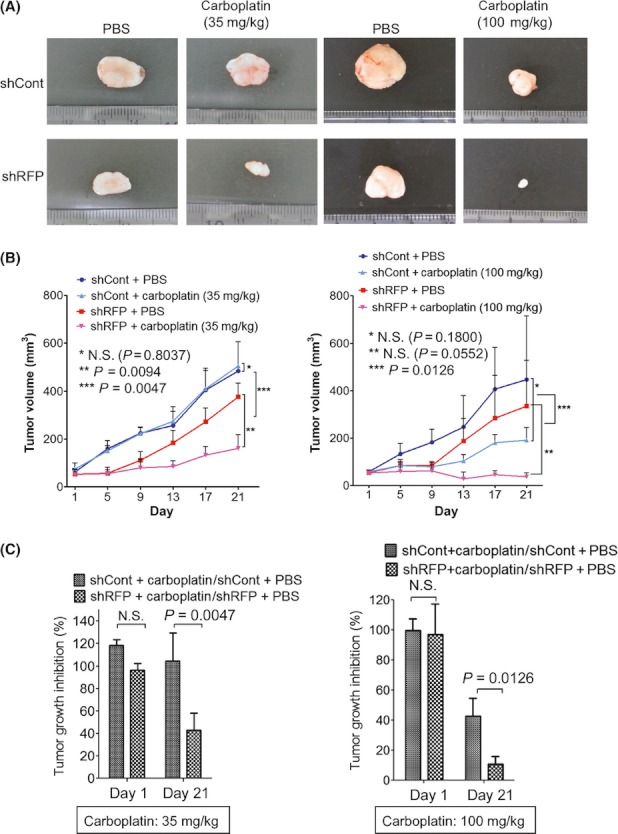
RFP knockdown enhances the sensitivity of tumors to carboplatin in vivo; 5 × 10^6^ SKOV3 ovarian cancer cells infected with retroviruses carrying shCont or shRFP were inoculated subcutaneously (s.c.) into nude mice. When tumors reached approximately 50 mm^3^, 10 mice were treated with phosphate-buffered saline (PBS) or carboplatin (35 or 100 mg/kg) i.p. four times every 2 days. (A) Representative transplanted tumors excised at day 21 are shown. (B) Tumors were measured at different days after PBS or carboplatin treatment, and growth curves were drawn. Tumor volume of the carboplatin-treated shRFP group was significantly smaller than that of the control group. Left, 35 mg/kg carboplatin treatment group; right, 100 mg/kg carboplatin treatment group. (C) The mean tumor volume of the PBS-treated group was defined as 100% and compared with that of the treated group on days 1 and 21. Statistic analysis was performed by Student's *t*-test. Carboplatin treatment group, *n* = 5; PBS group, *n* = 3; bars, SD.

To determine whether RFP knockdown influences the sensitivity of ovarian cancer cells to carboplatin in vivo, athymic nude mice were inoculated with 5 × 10^6^ SKOV3 cells expressing either shCont or shRFP. The mice were administered carboplatin at 35 or 100 mg/kg per day i.p. four times every 2 days. In the shCont group, treatment with 100 mg/kg per day of carboplatin significantly inhibited tumor growth, whereas treatment with 35 mg/kg per day had little effect (Fig. 5A and B). Treatment with both doses of carboplatin dramatically decreased tumor size in the shRFP group. By day 21, tumors from shRFP SKOV3 cells treated with carboplatin at 35 and 100 mg/kg per day resulted in a reduction of tumor volume by 56.9 ± 8.6% and 95.7 ± 1.6%, respectively (Fig. 5B and C). These results show that RFP knockdown in ovarian cancer cells confers higher carboplatin sensitivity in vivo.

## Discussion

Platinum/paclitaxel-based chemotherapy is the most common method for treating advanced ovarian cancer. The existence or development of chemoresistance is a critical factor for reducing the efficacy of chemotherapy. Therefore, the development of good predictive markers for chemoresistance is valuable for optimizing ovarian cancer treatment.

In this study, we investigated RFP expression in epithelial ovarian cancer using 92 surgical specimens and found that RFP expression significantly correlates with chemoresistance in ovarian cancer patients. In addition, positive RFP staining was correlated with disease recurrence. These findings suggest that RFP expression enhances the intrinsic primary chemoresistance in ovarian cancer. Although this study is retrospective and the patient number is limited, our findings could have potential clinical implications. Larger prospective studies are necessary in the future.

Consistent with clinicopathological analysis, RFP depletion by siRNA sensitized the ovarian cancer cell lines to carboplatin and paclitaxel through an increase in apoptosis. One possible mechanism by which RFP regulates carboplatin resistance is the regulation of TBP-2 expression. TBP-2 inhibits thioredoxin, a scavenger of reactive oxygen species (ROS), and sensitizes cells to oxidative stress and cisplatin [22–26]. We have recently demonstrated that RFP interacts with HDAC1 and confers platinum-based drug resistance in cancer cell lines, including colon, breast, and cervical cancer cell lines, by decreasing TBP-2 expression [19]. In that study, we also found that knockdown of TBP-2 was not able to fully increase platinum resistance decreased by RFP knockdown. This finding suggests the existence of other mechanisms in RFP-mediated platinum resistance. Reles et al. reported that the mutation status of p53 as well as mutant p53 overexpression was correlated with resistance to platinum-based chemotherapy in ovarian cancer [9]. Interestingly, RFP was shown to possess SUMO E3 ligase activity that targets p53 and MDM2 [27]. On the basis of our recent study, RFP can also bind to USP7, a p53-regulating ubiquitin-specific protease (Kato et al. unpubl. data). Taken together, these findings suggested that RFP may regulate carboplatin resistance through modulating p53 activity or expression.

In our experiment, RFP knockdown also decreased paclitaxel resistance. Sensitivity to paclitaxel is known to be increased in p53 knockout cells [28, 29]. Recently, Catuogno et al. reported that miR-34c downregulated p53 expression and increased the cancer cell's sensitivity to paclitaxel by suppressing Bmf expression [30]. Given the transcriptional regulation ability and possible p53-modulating role of RFP, RFP might confer paclitaxel resistance to ovarian cancer cells through direct regulation of p53 function or transcriptional regulation of certain genes, such as miR-34c. Further study is necessary to clarify the precise mechanisms of RFP-mediated chemoresistance.

Interestingly, while RFP knockdown did not affect the proliferation rate of cancer cell lines in vitro, shRFP-expressing cells exhibited a significant decrease in tumor growth in an athymic mouse xenograft model compared with shCont-expressing cells. This is probably because tumor cells inoculated into mice were exposed to more stressors such as defective nutrition and hypoxia than those in an in vitro culture environment [31, 32]. Thus, RFP may also render cancer cell resistance to various stressors other than oxidative stress. Indeed, we observed that RFP knockdown increases cellular sensitivity to UV irradiation in HeLa cells (our unpubl. data). This finding supports our speculation that RFP could regulate cellular sensitivity to anticancer drugs through mechanisms other than regulation of TBP-2 expression.

In conclusion, we demonstrated that RFP expression is correlated with chemoresistance in ovarian cancer patients. Furthermore, inhibition of RFP expression potently enhances the therapeutic effect of carboplatin and paclitaxel. Our results provide novel insight into the exploration of predictive markers for chemoresistance and/or effective therapeutic strategy for ovarian cancer.

## Supporting Information

**Table S1.** Association between RFP expression and platinum-free interval in four histological types of epithelial ovarian cancer.

**Table S2.** Multivariate analysis of several clinicopathologic parameters in relation to survival of patients with epithelial ovarian cancer.
