# Supplementary material for: Expression of RET finger protein predicts chemoresistance in epithelial ovarian cancer
Source: Cancer Med. 2012 Sep 13;1(2):218–29. doi: 10.1002/cam4.32 (PMC3544444; doi:10.1002/cam4.32)
Supplement: Supplementary file 1 [file cam40001-0218-SD1.doc]

Table S1: Association between RFP expression and platinum–free interval in four histological types of epithelial ovarian cancer

|  |  |  |  |  |
| --- | --- | --- | --- | --- |
| Time before recurrense (pletinum-free interval) | Number  of patiens | RFP(-) | RFP(+) | *P*-value |
| serous ≧6months | 32 | 17(53.1％) | 15(46.9%) |  |
| <6months | 13 | 2(15.4%) | 11(84.6%) | *0.0202* |
| micinous ≧6months | 5 | 2(40.0%) | 3(60.0%) |  |
| <6months | 5 | 2(40.0%) | 3(60.0%) | 1.000 |
| endometrioid ≧6months | 8 | 5(62.5%) | 3(37.5%) |  |
| <6months | 5 | 1(20.0%) | 4(80.0%) | 0.1348 |
| clear cell ≧6months | 15 | 6(40.0%) | 9(60.0%) |  |
| <6months | 9 | 0(0.0%) | 9(100.0%) | *0.0285* |
|  |  |  |  |  |

Table S2: Multivariate analysis of several clinicopathologic parameters in relation to survival of patients with epithelial ovarian cancer

|  |  |  |  |
| --- | --- | --- | --- |
| Variable | OS |  |  |
|  | Hazard ratio(95% CI) |  | *P* |
| Age |  |  |  |
| ≧60(*vs* <60) | 1.097 (0.535-2.248) |  | 0.800 |
| FIGO stage |  |  |  |
| II-IV(*vs* I) | 2.301 (1.023-5.175) |  | *0.044* |
| Histological type |  |  |  |
| clear cell (*vs* nonclear) | 1.053 (0.385-2.878) |  | 0.920 |
| RFP expression |  |  |  |
| positive(*vs* negative) | 1.441 (0.754-2.751) |  | 0.269 |
|  |  |  |  |

OS; Overall Survival P<0.05; Statistically significant
